# Synthesis of Metal–Organic Frameworks Quantum Dots Composites as Sensors for Endocrine-Disrupting Chemicals

**DOI:** 10.3390/ijms23147980

**Published:** 2022-07-20

**Authors:** Peter A. Ajibade, Solomon O. Oloyede

**Affiliations:** School of Chemistry and Physics, University of Kwazulu-Natal, Private Bag X01, Pietermaritzburg 3209, South Africa; 221118851@stu.ukzn.ac.za

**Keywords:** metal organic framework, synthesis, quantum dots, endocrine disruptors, bisphenols

## Abstract

Hazardous chemical compounds such as endocrine-disrupting chemicals (EDCs) are widespread and part of the materials we use daily. Among these compounds, bisphenol A (BPA) is the most common endocrine-disrupting chemical and is prevalent due to the chemical raw materials used to manufacture thermoplastic polymers, rigid foams, and industrial coatings. General exposure to endocrine-disrupting chemicals constitutes a serious health hazard, especially to reproductive systems, and can lead to transgenerational diseases in adults due to exposure to these chemicals over several years. Thus, it is necessary to develop sensors for early detection of endocrine-disrupting chemicals. In recent years, the use of metal–organic frameworks (MOFs) as sensors for EDCs has been explored due to their distinctive characteristics, such as wide surface area, outstanding chemical fastness, structural tuneability, gas storage, molecular separation, proton conductivity, and catalyst activity, among others which can be modified to sense hazardous environmental pollutants such as EDCs. In order to improve the versatility of MOFs as sensors, semiconductor quantum dots have been introduced into the MOF pores to form metal–organic frameworks/quantum dots composites. These composites possess a large optical absorption coefficient, low toxicity, direct bandgap, formidable sensing capacity, high resistance to change under light and tunable visual qualities by varying the size and compositions, which make them useful for applications as sensors for probing of dangerous and risky environmental contaminants such as EDCs and more. In this review, we explore various synthetic strategies of (MOFs), quantum dots (QDs), and metal–organic framework quantum dots composites (MOFs@QDs) as efficient compounds for the sensing of ecological pollutants, contaminants, and toxicants such as EDCs. We also summarize various compounds or materials used in the detection of BPA as well as the sensing ability and capability of MOFs, QDs, and MOFs@QDs composites that can be used as sensors for EDCs and BPA.

## 1. Introduction

### 1.1. Metal–Organic Frameworks

**Figure 1:**
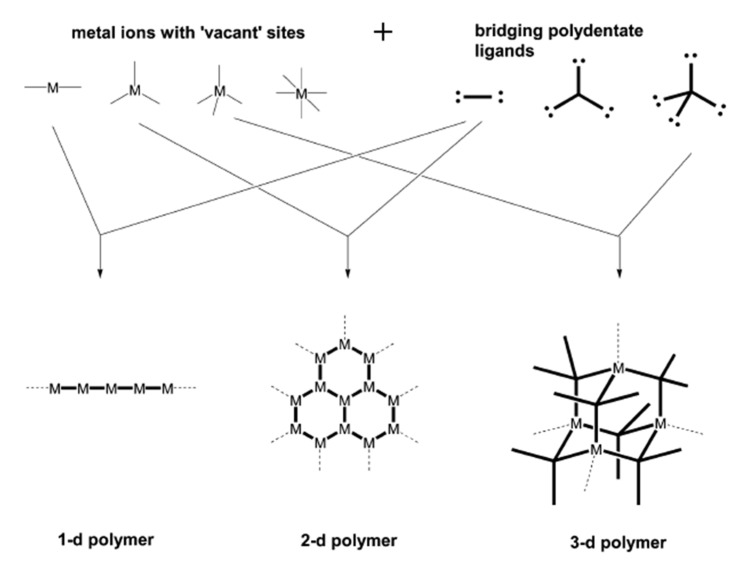
Building block principle behind formation of coordination polymers.

Coordination polymers are chemical architectures constructed through the coordination of metal ions and organic linkers through integrated chemical bonds. They are solids state structures with repeated coordination units that could be one, two, or three dimensions [1] (Figure 1). They are described as polymers whose repeating units are coordination composites. Coordination polymers include the subcategory coordination matrix, which are coordination compounds expanding through continual coordination entities. A subclass of these is metal–organic frameworks (MOFs), which are coordinated to organic linkers with potentials vacuum [2].

**Figure 2:**
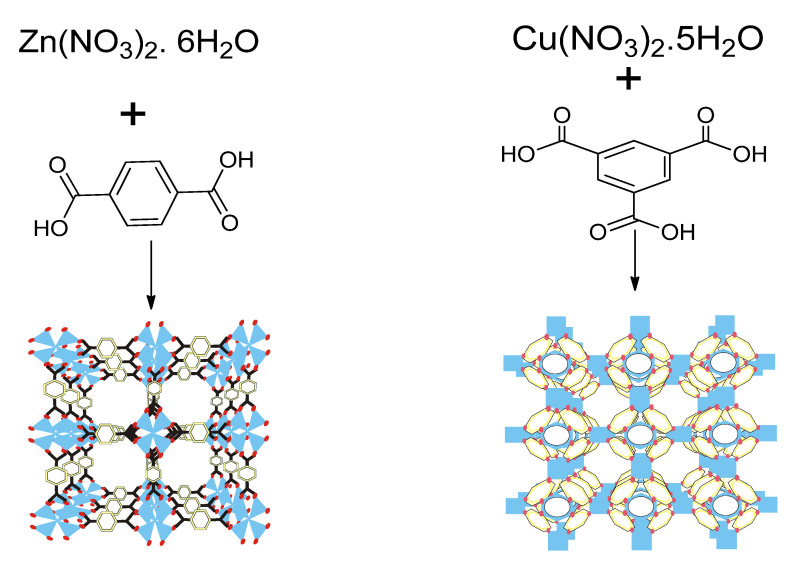
Typical synthesis of metal–organic frameworks.

**Figure 3:**
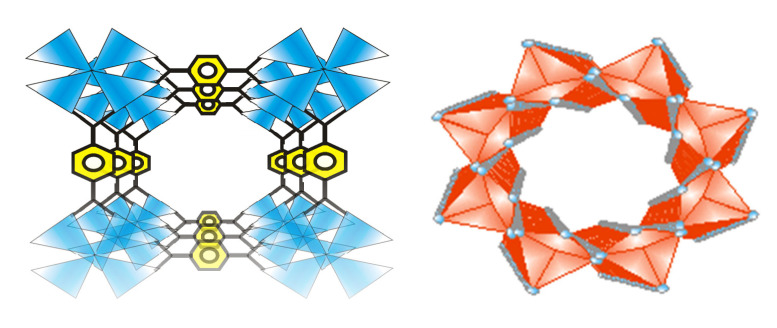
Typical structures of metal–organic frameworks with different metal salt and linkers.

Metal–organic frameworks are functional spongy coordination polymers with a crystal-like structure comprised of metal ions and cluster linked by polydentate organic ligands (Figure 2). Metal–organic frameworks have exceptional structural features (Figure 3), such as large surface area, large pore sizes, large pore volumes, diverse topologies, ease of functionalization, controllable synthesis, flexibility of the pore sizes, and thermal stability [3].

**Figure 4:**
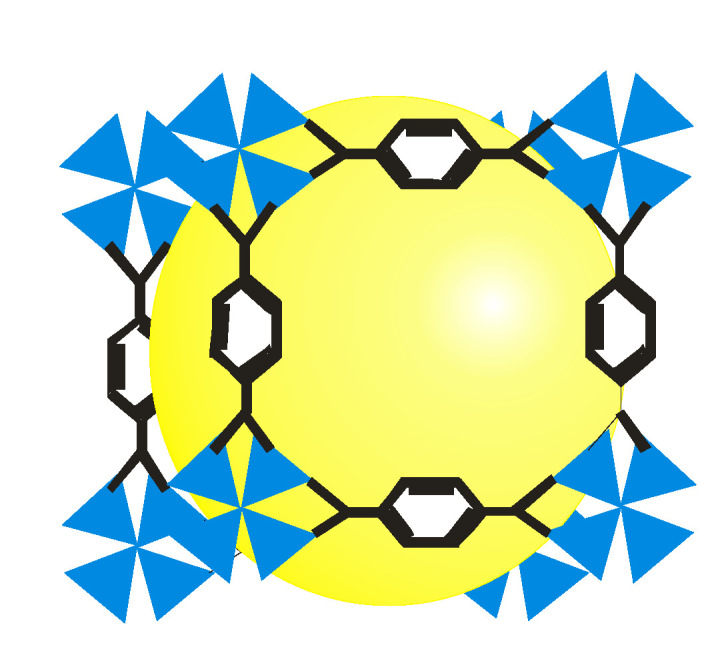
Structure of typical metal–organic frameworks used for gas storage.

Research on the synthesis, properties, and application of MOFs is due to their structure and their pore volume, which can accommodate different kinds of nanoparticles to form MOFs/nanoparticles composites which can be tailored for different applications [4]. MOF composites have unique structural features and inherent semiconductor properties, which make them good materials for different applications such as gas storage (Figure 4), gas separation [5,6], gas purification, heterogeneous catalysts [7,8], adsorbents [9,10,11], ions exchange, microelectronics, magnetism, drug loading, drug delivery [12], molecular recognition, biomedicine, bio-imaging, and luminescence [13].

**Figure 5:**
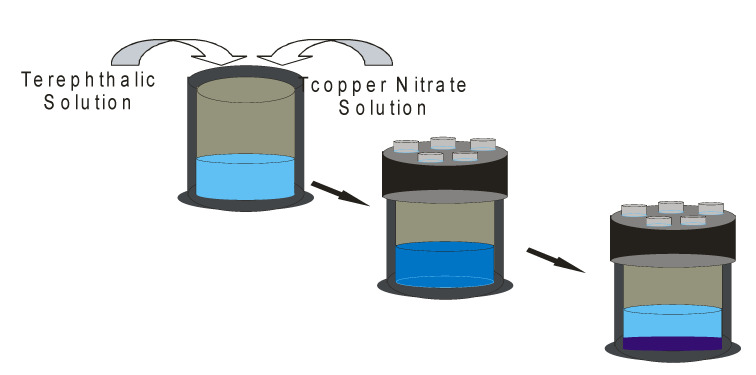
Schematic representation of solvothermal synthesis of MOFs.

MOFs have been prepared using different solvent-based techniques, such as solvothermal in which the compounds are prepared under reflux at the boiling point of the solvent, and mechanochemical synthesis, which is a solvent-free synthetic technique. The two approaches are regarded as the conventional and unconventional methods of preparations for metal–organic frameworks [14]. Another example of solvothermal synthesis is the autoclave synthesis in which the reactants are sealed into an autoclave and in turn transferred to the oven at the temperature below the boiling point of the solvent (Figure 5).

In recent years, rapid pollution has resulted in the discharge of several harmful pollutants into ecological ecosystems, affecting public health. These pollutants are diverse and include trace metals, organic compounds, and deadly gases [15]. Thus, it is necessary to develop sensors that can be used to detect these harmful chemicals in the environment [16]. The presence of a sensing entity and a transduction part in a detector contribute to the translation of the sensed information into different type of signals, which can be electrical or optical wave [17,18]. The mechanism of a detector is majorly rooted in its transduction which is related to the attributes of the detector with regard to its electrical, mechanical, photophysical, optical features when it interacts with analytes [19,20,21].

A good sensor should be quick to respond, firm, selective, sensitive and be reusable [22]. Thus, in the design of sensors, the composition or the types of materials used determine its effectiveness and both microscopic and macroscopic materials have been used to fabricate ecological monitor sensors. [23]. These materials include carbon nanotubes, graphene [24,25,26,27,28], metals and metal oxides [29,30,31], semiconductor materials [32,33], quantum dots [34,35] and polymers [36,37]. The preparation and understanding of the unique properties of these materials have generated great interest in the development of new sensors with novel applications.

**Figure 6:**
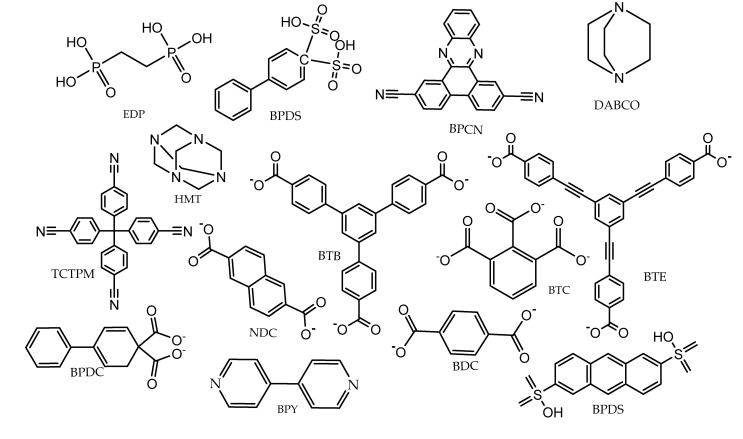
Typical ligands used for MOF synthesis.

MOFs, with their diverse architecture, tunable chemical functionalization, reversible adsorption, and high catalytic activities with respect to the ligands employed (Figure 6), are attractive chemical entities for the development of chemical sensors [38]. MOFs have been designed and functionalized to selectively detect and adsorbed contaminants such as trace metals, poisonous gases, and hydrocarbons in the environment [39].

### 1.2. Quantum Dots

**Figure 7:**
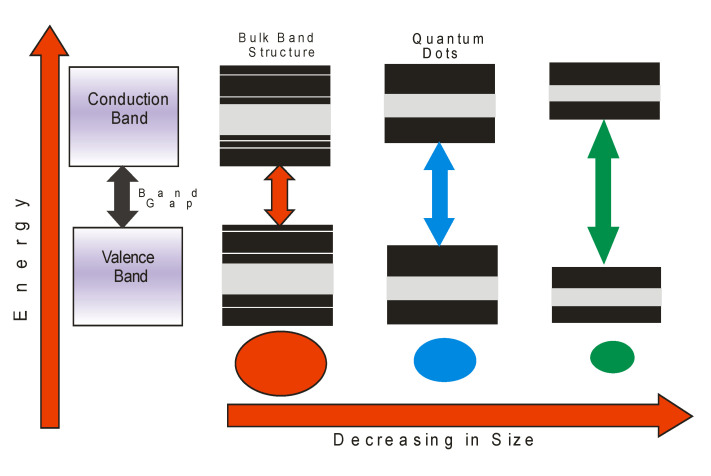
Quantum dots band gaps relative to particle sizes.

**Figure 8:**
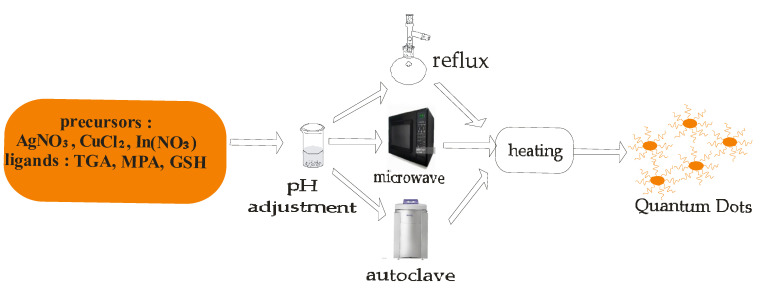
Synthetic scheme for quantum dots in aqueous medium.

Semiconductor quantum dots have received tremendous attention due to their unique physical, chemical, optical, and electronic properties which make them useful in wide ranging and novel applications [40,41,42]. Quantum dots (QDs) are zero dimensional materials that are quantum confined in all three dimensions with particle sizes in the range 1–10 nm; their properties can be tuned through engineering of shape- and size-dependent morphologies for specific applications [43,44]. Their unique optoelectronic properties such as tunable energy band gaps (Figure 7), high absorption coefficients, and exciton generation are useful for the development of photovoltaic devices [45,46]. Quantum dots fabricated from II–VI and III–V compound semiconductors have physical sizes that are smaller than the Bohr exciton radius with energy band gaps that reflect different colors based on the particles in the visible region of the electromagnetic spectrum [47,48,49,50]. Semiconductor quantum dots were some of the first nanomaterials to be used for biomedical applications, with the cadmium selenide/zinc selenide core shell being used as secondary antibody conjugates for diagnostic and therapeutic applications [51,52,53]. Semiconductor quantum dots can be prepared using different techniques in aqueous medium (Figure 8).

### 1.3. Endocrine-Disrupting Chemicals

**Figure 9:**
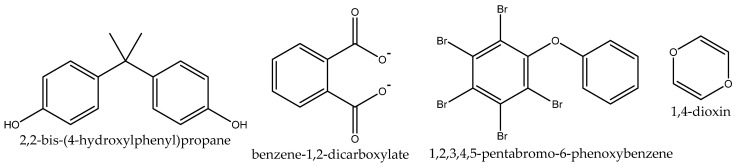
Example of endocrine-disrupting compounds.

The endocrine and nervous systems work together to control body functions, movement and steroid hormones in the human brain through regulated and coordinated processes, but these can be disrupted by the presence of external substances that inhibit the functions of natural hormones and make them vulnerable [54]. Common exogenous compounds that can interact with the endocrine systems are endocrine-disrupting chemicals (EDCs), which are public health problems. These common exogenous compounds include plasticizers, flame-retardants, fungicides, pesticides, pharmaceuticals, and heavy metals. Other naturally occurring compounds, such as phytoestrogen-containing endocrine-disrupting compounds are bisphenol A, chlorpyrifos, dioxins, triethyl lead, methoxychlor, phthalates (benzyl butyl phthalates), pentabromodiphenyl ether, and vinclozolin (Figure 9) [55].

#### Bisphenol A (BPA)

**Figure 10:**
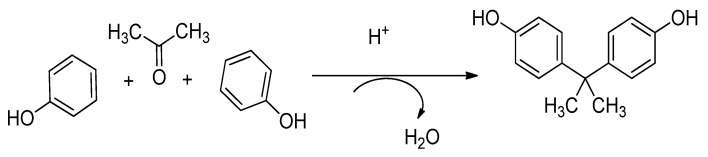
Synthesis and chemical structure of bisphenol A.

Bisphenol A (2,2-bis (4-hydroxyphenyl) propane) is a chemical used primarily as a macromolecule in the production of polycarbonate plastics (PC) and adhesives. It has also been used in polyesters, polysulfide, homopolymers and flame retardants. Polycarbonates are used to manufacture household and retail food packaging. It can be prepared in the laboratory as shown in Figure 10. The protective linings of most canned food and beverages are made from epoxy resins and a vanish layer on metals caps for glass jars and bottle, which leads to bisphenol A (BPA) [56]. BPA was established in the 1930s to bind to steroids receptor but its potential use as pharmaceutical was abandoned and replaced with diethyl stilboestrol, a much more potent estrogen [57]. It has been established that high levels of BPA in males leads to reduction in semen [58]. Studies have shown that increases in the levels of BPA result in reduced sexual drive, erectile dysfunction, and ejaculation inconveniency in some men. It has also been shown that these are associated with reduction in semen quality as a result of recent exposure to BPA [59].

Recent studies of cohorts of 84 [60] and 147 women [61] undergoing IVF treatment showed that the number of oocytes retrieved from these women depends on their urinary BPA concentration. These results revealed that oocyte maturation and fertilization could be affected by exposure and concentration of BPA, which impairs oocytes during IVF, which in turn affects the success or otherwise of the IVF treatment. Endocrine-disrupting chemicals such as BPA have been shown to cause neurobehavioral disorders such as autism spectrum disorder (ASD) and attention deficit/hyperactivity disorder [62].

### 1.4. Heavy Metals

Naturally occurring elements with a density and atomic weight at least five times greater that of water are called heavy metals. These elements are used in all sectors of life (medical, industrial, domestics, technological, agricultural), have led to widespread discharge in the environment and constitute dangers to human health and the environment. Their speciation, route of exposure, and the type of metal ions determines their level of toxicity [63]. These heavy metals include arsenic, cadmium, chromium, and lead, among others, and are regarded as systemic toxicants that can induce multiple organ destruction at very low concentrations. Metal corrosion, atmospheric deposition, soil erosion, leaching, sediment re-suspension, and evaporation from water of these heavy metals are major sources of these pollutants in the environment [64].

## 2. Metal–Organic Frameworks

### 2.1. Metal–Organic Frameworks as Potential Sensors for Pollutants in the Environment

Metal–organic frameworks (MOFs) possess many exciting characteristic features, such as flexibility, ordered crystalline pores and multiple coordination sites, which makes them versatile materials as sensors for environmental pollutants and toxicants [65,66,67,68]. Sha et al. [69] designed a ratiometric fluorescence sensor based on metal organic frameworks and ruthenium bipyridyl doped silica composites for the detection of 17β-estradiol. Streptavidin was restrained on the fabricated metal–organic frameworks (MIL-53-NH_2_) by covalent connection and further coupled with the biotin-improved aptamer through definite bonding between avidin and biotin. This sensitive fluorescence sensor could detect the most active 17β-estradiol in mammalian estrogen steroids. The fabricated sensor could achieve delicate detection of E2 due to a great overlay between the absorption spectrum of its acceptor (RuSiO_2_-cDNA) and the radiation spectrum of the FRET donor (MIL-53 apt). The choice of the green non-toxic nanoparticles and aptamer improved the sensitivity to detection by the sensor through dual signal detection. The limit of detection of 0.2–0.3 μM was recorded in goat serum with good reproducibility and selectivity [69].

Jin et al. [70] synthesized an ultraselective and ultrasensitive electrochemiluminescence (ECL) aptasensor based on metal–organic frameworks (Co-Ni/MOF) with perylene (BP/PTC-NH_2_)/peroxydisulfate (S_2_O_8_^2−^) that was used as sensor for choramphenicol (CAP). The nanocomposites were synthesized by dissolving black phosphorus quantum dots into the precursor solution of perylene derivative (PT-NH_2_). The Co-Ni/MOF, which has excellent encapsulation and an effective catalytic property, was added in order to react with the co-reactant (K_2_S_2_O_8_) to produce more SO_4_^2−^, thus raising the indicator of the compound 3.8-times higher than that of the single BP/PTC-NH_2_. The as-synthesized nanocomposites sensor Co-Ni/MOF/BP/PTC-NH_2_/K_2_SO_4_^2−^ shows high electrochemiluminescence activity and could accurately be used to detect choramphenicol. In the presence of chloramphenicol an enhanced signal was observed, ascribed to the aptamer concession, which caused the aptamer to pull from the sensing interface by choramphenicol. Under ideal conditions, the concentration of choramphenicol can be quantified by the aptasensor from 1.0 × 10^−13^ M to 1.0 × 10^−6^ M with low limit of detection of 2.9 × 10^−14^ M [70].

Chen and co-workers [71] developed a composite of Ƴ-Fe_2_O_3_ at high temperature and used it as sensor for hydrogen sulfide (H_2_S) at ambient temperature. The composite was obtained by dispersion of Ƴ-Fe_2_O_3_ octahedron obtained from MIL-88 on reduced graphene oxide (rGO). This composite, Ƴ-Fe_2_O_3_/rGO, had an enhanced gas-sensing performance in comparison to the Ƴ-Fe_2_O_3_ sensor, ascribed to the reinforcement or synergistic properties of the Ƴ-Fe_2_O_3_/rGO which combined the properties of reduced graphene oxides and the iron(III) oxides. The Ƴ-Fe_2_O_3_/rGO composite is highly sensitive for the detection of hydrogen sulphide (H_2_S) gas at room temperature with opposition of the sensor to fresh air and aim gas to be 520.73, 97 ppm (Rair/Rgas = 520.73 97 ppm). The sensing ability of the composite is to the bulk resistance effect of the iron(III) oxide (Ƴ-Fe_2_O_3_) on the surface of the reduced graphene oxide (rGO). The sensing ability of the composite Ƴ-Fe_2_O_3_/rGO (iron(III) oxide/reduced graphene oxide) to act as efficient sensor for hydrogen sulfide is a result of the outstanding conductivity and large active sites of the two-dimensional reduced graphene oxide. However, the composites could not be used as sensors for other trace toxic gases such as NH_3_, CHCl_3_, NO, SO_2_, HCHO [71].

One of the sensitive and specific biomarkers for sudden cardiac arrest is N-terminal pro-brain natriuretic peptide (NT-proBNP). Surface-enhanced Raman spectroscopy (SERS) can precisely detect NT-proBNPs at an early stage for management. Ye He et al. established a new SERS-based immunosensor for ultrasensitive scrutiny of NT-proBNPs by using a MOF complex isoreticular metal–organic framework gold tetrapod (IRMOF@Au-tetrapods). MOFs@AuTPs immobilized toluidine blue as SERS tag. MOF complex MOF@AuTPs were utilized for immobilized antibody and Raman probes for their brilliant characteristics of high absorbency and easily fixed biomolecules because of its biocompatibility and large surface area. The detection of NT-proBNPS was achieved using a magnet/metal–organic framework. This SERS-based immunosensor has many advantages such as simplified analysis (using magnetic CoFe_3_O_4_, small ingesting (10 µL), and high sensitivity [72].

**Figure 11:**
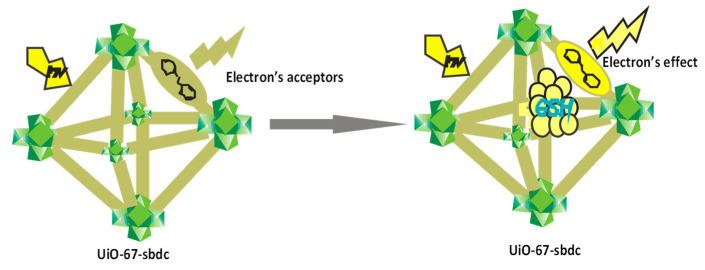
Architecture and proposed mechanism of UiO-67-sbdc to GSH.

Glutathione (GSH) is a tripeptide that contains cysteine, glycine, and glutamine and is found in the human body as an antioxidant that protects the body against toxins and also prevents damage to the body cells from reactive oxygen caused by free radicals [73,74]. Any sharp decrease of GSH in the body fluid can be as a result of harmful diseases such as cancer, Alzheimer’s disease, or HIV. The maintenance of the amount of GSH in the body fluid is of high significance. Research to promptly detect the amount of GSH for its clinical importance is very crucial [75,76,77,78]. Jiayan et al. [79] synthesized a fluorescence sensor metal–organic framework UiO-67-sbdc (MOF) for the detection of GSH, which is a key factor in redox reactions in biological process (Figure 11). The automatic effect between GSH and the ligand of MOF effectively enhanced the florescence of MOF to be able to detect GSH. Powder X-ray diffraction (PXRD), scanning electron microscopy (SEM), transmission electron microscopy (TEM), Fourier transform infrared spectroscopy (FTIR), and energy dispersed X-ray spectroscopy (EDX) were used to characterize the as-synthesized MOF. Upon addition of GSH solution, the fluorescence of MOF showed a sharp response which indicates that the MOF can work as a fast reaction fluorescence sensor for GSH in medical diagnostic tests. This rapid response of the MOF is ascribed to its high surface area and its porous structure (13.5 Å). The size of GSH synthesized is small for the pore channel of UiO-67-sbdc (13.5–13.5 Å), which helps with the diffusion of the nanoparticles into its microscopic pores with little opposition and deepens the analyte in spongy assembly, thus increasing its exposure to functional sites and boosting the detection process. Serum was also used for the testing of the fluorescence response of UiO-67sbdc to GSH; the detection limit was projected to be 107.2 µM with a suitable linear range [80].

The use of different chemicals as food additives has become a public health hazard and some of these chemicals, such as clenbuterol, mycotoxins, heavy metals and organic pollutants, have become widely distributed in ecosystems. For instance, mycotoxins such as deoxynivalenol (DON) and salbutamol (SAL) are very harmful to both human and animals because they inhibit the production of DNA, RNA and proteins by clinging to the ribosomal peptidyl transferase and causing serious sickness [81]. A novel Co-Ni based bimetallic metal–organic framework was synthesized by Yingpan et al. [82] with the use of a mixed ligands of 4-(1H-tetrazol-5-yl)benzoic acid (H_2_TZB) and 2,4,6-tri(4-pyridyl)-1,3,5-triazine (TPT) to detect these harmful substances in the environment. Structural and morphological studies showed that the bimetallic MOF are electrochemically active than the singular Co-MOF and Ni-MOF. *Van der Waals* forces, non-covalent interactions between carboxyl groups of H_2_TZB and amino groups of antibodies [83,84], enables the antibodies to bind to the surfaces of deoxynivalenol, and salbutamol results in the efficient response of the materials as sensors through electrochemical interactions. This electrochemical-based immunosensor Co-Ni-MOF showed a better performance compared with the individual metal–organic framework of cobalt and nickel, with a detection limit of 0.05 pg·mL^−1^ and 0.30 pg·mL^−1^ towards deoxynivalenol (DON) and salbutamol (SAL), respectively, in the concentration range of 0.001 to 0.5 ng·mL^−1^. The bimetallic MOF has unique properties such as regenerability, selectivity, stability, and reproducibility, and can easily be used for practical applications [82].

**Figure 12:**
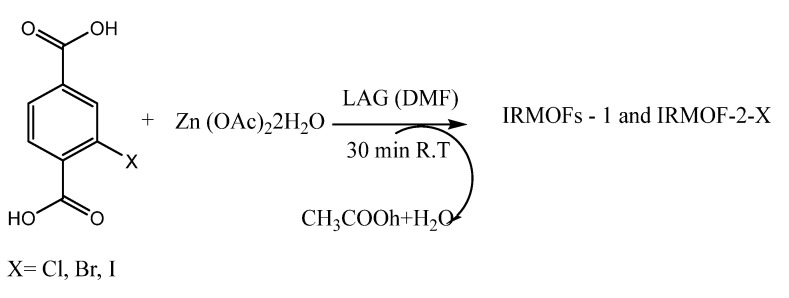
Mechanochemical synthesis of IRMOF-1 and IRMOF-2-X (X = Cl, Br, I).

**Figure 13:**
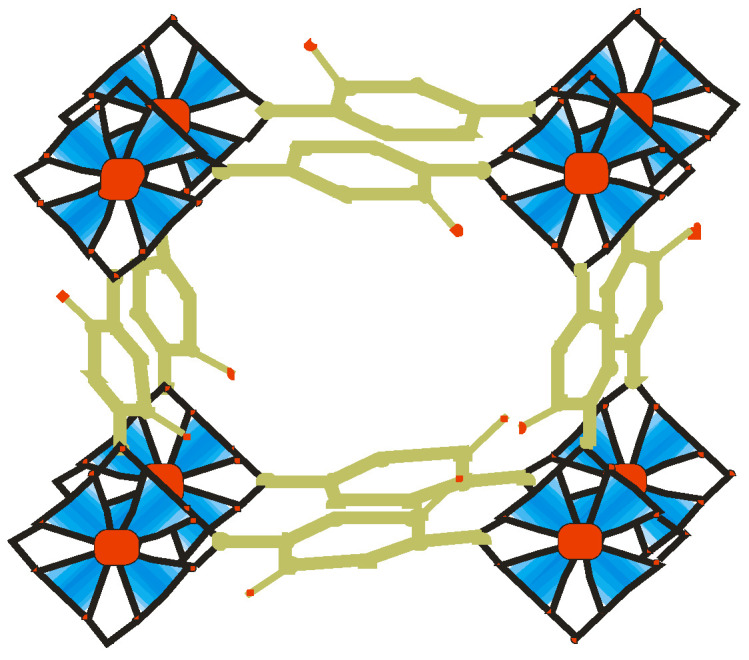
Representation of the structure of IRMOF-1 and IRMOF-2-X.

The sensing and elimination of poisonous chemicals compounds such as polycyclic aromatics hydrocarbons (PAHs) [85] and nitroaromatic compounds (NACs) [86] have been studied using different MOFs. Among these MOFs, an identical isoreticular metal–organic framework (IRMOF) provides a series of opportunities for researchers to thoroughly explore the effect of the organic linkers on the performance of the molecules as sensors [87]. In this regard, Sedigheh et al. [88] report the synthesis of IRMOF-1 and IRMOF-2-X to examine the importance of the halogen atoms on MOF interactions. In addition to the lack of early reports on these IRMOFs, the use of these compounds as sensors for the detection of nitrobenzene has been studied. These researchers synthesized IRMOFs-1 and IRMOFs-2-X (Figure 12 and Figure 13) via a liquid assisted grinding (LAG). The result of the experiment revealed a quenching observation of the effective fluorescence for nitrobenzene (PhNO_2_), which is a known poisonous explosive contaminant. More than 95% quenching efficiency was reached for nitrobenzene, and the importance of the functionality of IRMOF-1 and its sensing properties were explored. The Stern–Volmer equation was used to monitor the IRMOFs, which showed that the value of Kq for IRMOF-1 was 508.14 M^−1^, which is beyond 10-times greater than Kq for the halogenated IRMOFs: IRMOF-2-Cl, IRMOF-2-Br, IRMOF-2-I, which are 7.75 M^−1^, 34.25 M^−1^, 30.35 M^−1^, respectively [89]. Since the inductive effect is strongly deduced by the electron transfer quenching, the order of the observed fluorescence quenching follows IRMOF-2-Br > IRMOF-2-I > IRMOF-2-Cl; the electron deficiency of the nitro group in the analyte and electron-donating characteristic, as well as the polarizability of the fluorophore in this series of halogenated IRMOFs, could be firmly responsible for these series [90,91,92]. Table 1 shows some metal-organic frameworks that are being explore as sensors for pollutants.

Environmental concerns about the use of explosive and explosive-like chemical substances and their proliferation in the modern world necessitate the development of sensors to detect these chemicals in the environment [93]. Current methods of detection of these compounds typically involve canine or other sophisticated instruments. Several countries cannot afford the implementation of these methods because of the high cost. Florescence-quenching techniques that use interfused polymers based on the donor and acceptor electron process are cost-effective techniques that are being developed to detect these chemicals [94,95,96]. Metal–organic frameworks (MOFs), which are porous coordinated compounds synthesized with conjugated ligands, are outstanding electron donors, and the delocalized π* excited state enhances their abilities to donate these electrons, which facilitate exciton movement and hence intensifies the electron cooperation between the polymers and electron defective nitroaromatic analytes [97,98]. Pramanik et al. synthesized a highly luminescent MOF [(Zn_2_(oba)_2_(bpy).DMA] (H_2_Oba = 4,4-oxybis(benzoic acid); bpy = 4,4-bipyridine; DMA = N,N′-dimethylacetamide]. The selection of this MOF by the authors is credited to the reported electronic features of the MOF as well as the outcomes of the molecular orbital, which is based on the interactions of electrons in the excited state, the electronic band framework calculation, the electrochemical measurements, and the high sensitivity ability of detection of the nitroaromatic compounds in their vapor phases [99]. The results of the study indicate that the electron-withdrawing nitroaromatic compounds act as fluorescence quenchers with nitrobenzene being the most effective with quenching efficiency of 84% and dinitrotoluene the least effective. The luminescence emission of the MOF appeared to be enhanced by the non-nitro-containing aromatics. Toluene enhances the emission of the MOF the most by 120% while chlorobenzene is the least of the group. The techniques of donor–acceptor electron transfer between the analyte and the MOF could be ascribed to the observed enhancement [100].

### 2.2. Quantum Dots as Sensors for Environmental Pollutants and Other Toxic Substances

**Figure 14:**
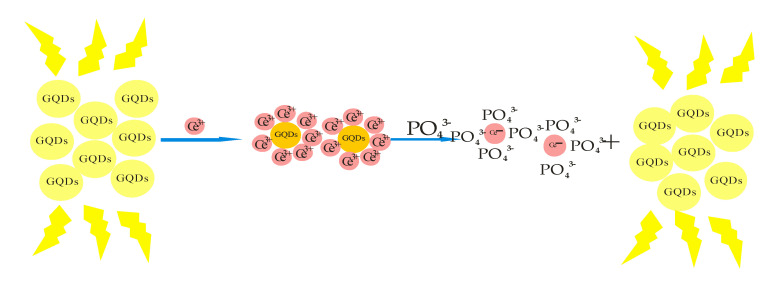
Design of phosphate sensors based on GQDs/Ce^3+^ nanoparticles.

Table 2 below summarizes some quantum dots as sensos for environmental pollutants and other toxic substances discussed in the text. The unique electronic properties and two-dimensional carbon structure of graphene oxide (GO) has generated renewed research interest [101,102]. Interest in graphene oxide is due to its chemical reduction properties, which can be tuned based on the size and shape of the fabricated materials. Materials are referred to as graphene oxide quantum dots (GQDs) when the synthesis of GO sheets is smaller than 100 nm, and they possess robust quantum confinement effects due to their size-dependent properties that can be used to fabricate different opto-electronic devices [103]. Bai et al. synthesized photoluminescence graphene quantum dots for the detection of phosphate ion [PO_4_]^3−^, one of the main nourishments in the food chain of marine microorganisms and a suitable pointer of organic pollutants in the water body. In order to control and guard against eutrophication, it is important to be able to sense and detect phosphate ion [PO_4_]^3−^. Graphene sheets have been used as precursors for the preparation of GQDs using the hydrothermal approach [104]. The detection mechanism followed the reaction of GQDs and Eu^3+^ ions. Primarily Eu^3+^ can be linked to the carboxylate groups on the surface of the GQDs where they act as a connection for the initiation of GQDs accumulation. Consequently, the photoluminescence of GQDs is quenched through energy transfer process. Then due to the extraordinary affinity of Eu^3+^ ions for the oxygen donor atoms of phosphate ion [PO_4_]^3−^ compared to the carboxylic group on the surface of the GQDs, the grouped GQDs dissociated at the introduction of phosphate ion [PO_4_]^3^ [105,106]. In this case, the redispersion of GQDs results in the restoration of photoluminescence (Figure 14). The practical approach to phosphate ion [PO_4_]^3−^ detection has been carried out using artificial wetlands (Aws) which contained various contaminants such as heavy metal ions and anions such as phosphates [107]. An average of 15 µM and 37 µM from four determinations was realized for the samples studied; 96% and 83% of GQDs were recovered for the water samples [108].

One of the essential elements for the synthesis of hemoglobin in human systems is copper. The United States Environment Protection Agency (EPA) has therefore legalized the limit of copper in drinking water to 1.3 ppm [114]. However, numerous problems emerge when people are continually exposed to a high concentration of this metal ion. Various approaches such as atomic absorption spectroscopy (AAS) and inductively coupled plasma mass spectrometry (ICPMS) have been used to quantify the concentration of copper in drinking water. Due to the high cost of these methods, and the non-precise nature and complex detection process, Rajendran et al. synthesized water-soluble ternary CuInS_2_/ZnS quantum dots (QDs) coated by glutathione (GSH) for the sensing and detection of copper ions in solution. The GSH capped CIS and CIS/ZnS core/shell QDs were fabricated as reported by Jose et al. [115].

Photostability and pH analysis reveal that 70% of the initial fluorescence of the nanoparticles was maintained after some time of irradiation while the fluorescence emission was stable within pH of 4–11. The selectivity and sensitivity of the mechanism reveal that the quantum dots show a rapid repost in 3 min and were steady up to 25 min at the addition of copper ions. The quenching of fluorescence was perceived in the concentration range from 0–70 nM with a detection limit of 63 nM. Dynamic light scattering (DLS), high resolution transmission electron microscopy (HRTEM), Fourier transform infrared, and photoluminescence spectroscopy (PL) analyses reveal that the accumulation of QDs due to the complexation of copper ions with the GSH capping group of the QDs could be accountable for the quenching of the ternary quantum dots [109].

Glutathione, composed of glycine, cysteine, and glutamine, an antioxidant in living cells, is responsible for the defense of vital cellular constituents by protecting them against free radicals such as oxygen, nitrogen and poison species. A rise in the level of glutathione may boost defense mechanisms of not only normal cells but likewise cancerous cells and can lead to cardiovascular diseases [116]. Consequently, a standard level of GSH is vital in maintaining the health of the body system with regard to liver-damaging illnesses such as cancer, neurodegenerative illnesses, and human immune deficiency virus. Rasoulzadeh and Amjadi prepared luminescent water-soluble AgInS_2_ quantum dots via a green and simple hydrothermal method and used the as-prepared compound as sensors for glutathione assay [117]. In the preparation of the quantum dots, sodium periodate (NaIO_4_) in alkaline solution was introduced in order to enhance its chemoluminescence. The effect of NaIO_4_ concentration on the chemoluminescence (CL) intensity was scrutinized in the range of 3 × 10^−4^ to 2.5 × 10^−3^ mol/L. NaIO_4_ solution concentration of 1.6 × 10^−3^ mol/L was used to achieve the maximum CL signal. This maximum CL process can be mostly expanded by the energy transfer between excited singlet oxygen dimol, AIS QDs and electron-hole injection [118]. The upsurge of CL intensity upon the addition of GSH to the AIS QDs-NaIO_4_ CL system could be determined by the use of CL system for GSH assay. This is described by the interaction between GSH and the sodium periodate which steers the production of enough amounts of reactive oxygen species and so leads to the higher CL intensity. At the end of the study, a log-log linear relationship (R^2^ = 0.994) was obtained with the regression equation of log (ΔI) = 0.47 log C + 7.20 (ΔI = I − Io, I and Io represent the pointers in the existence and absence of GSH respectively, C is the concentration of the GSH in mol/L). The detection limit was calculated to be 2.8 × 10^−10^ mol/L, and the Relative Standard Deviation (RSD) was 3.1% for five replicable measurements of the analyte.

Uranium is one of the toxic heavy metals that is highly poisonous and radiologically dangerous. This harmful chemical is widely employed for industrial application as a fuel source for nuclear power plants, nuclear weapons, and medical purposes. The most stable oxidation state and soluble form with maximum bioavailability of this metal in the environs is uranyl ion (UO_2_^2+^). Due to its long half-life and high level of toxicity, uranium affects human health with severe ailments such as damaging the liver, lungs, skeleton, and kidneys. A total of 130 nM is the maximum concentration of uranium that should not be exceeded in drinking water [110,119,120,121]. Consequently, a proficient and quick method to sense and detect this toxic metal in trace concentrations is necessary. Good sensitivity and satisfactory analytical methods such as Inductively Coupled Plasma-Mass Spectrometry (ICP-MS) and Inductively Coupled Plasma-Optical Emission Spectrometry (ICP-OES) have been used but are highly expensive, require highly skilled operators, are time consuming, and have cumbersome sample preparation that needs to be replaced by easy and environmentally friendly methods [122,123,124]. Sadeghi et al. [125] synthesized CdSe quantum dots using green precursor material with the support of urea-thioglycolic acid-choline chloride ternary deep eutectic solvent (DES-CdSe QDs) via a developed single step hydrothermal approach. Deep eutectic solvent (DES), due to its unique properties such as low cost, low volatility, low toxicity, non-flammability, and biodegradability, has surfaced as a new rank of green with encouraging applications in various fields such as in sensing [126,127]. The synthesis of the TDES-CdSe QDs was prepared using the methods of Wang et al. [128] with some modification. After the experiment, the fluorescence quenching of TDES-CdSe quantum using metal ion could be elucidated by five processes including ligand competition, electron transfer, cation-exchange, inner filter effect, and binding to surface ligands. The fluorescence quenching process of TDES-CdSe to uranyl ions was explained by the performance of some experiments. There was a detection of maximum fluorescence intensity of TDES-CdSe at 560 nm without a red or blue dislocation of peak wavelengths in the presence and absence of uranyl ions which specifies that the fluorescence of TDES-CdSe is suppressed without electron transfer systems or the cation-exchange. According to the Langmuir plot obtained in the experiment it was observed that uranyl ions are fastened to the surface of the ligand. Based on these outcomes it was suggested that the quenching of the fluorescence intensity of TDES-CdSe may be due to the fact that TDES on the surface of the QDs were fastened by uranyl ions which then transfer electrons from TDES (donor) to uranyl ions (acceptor), thus reducing the uranyl ions. The functioning of the TDES-CdSe probe was enhanced based on the cantered central complex design. Under ideal conditions, the TDES-CdSe quantum probes provide a direct response to uranyl ions in the concentration range from 10–50 nM with a detection limit of 5.7 nM. The Relative Standard Deviation percentage (RSD%) of the developed method for five replicates of determination of uranyl ions at 100 nM was evaluated to be 1.9%.

A class of naturally occurring toxic substances that are of public concern worldwide are mycotoxins [111]. Aflatoxin 1 (AFB1), which is one of the aflatoxin families, is a minor metabolite of mycotoxins with similar structure. Aflatoxin 1 is the most poisonous, owing to its capacity to cause a large increase in the risk of liver cirrhosis, carcinoma in human beings and animals, and necrosis through the hindrance of RNA synthesis by the cell [129,130]. Lu et al. [131] prepared a target-focus switch on fluorescence aptasensor for minute Aflatoxin 1 (AFB1) measurements based on highly fluorescence ternary quantum dots (CdZnTe QDs) using Fluorescence Resonance Electron Transfer (FRET) principle. FRET could be depicted through intermolecular dipole–dipole coupling of non-radiative energy transfer from energy donors to acceptors [132]. The use of a tertiary quantum dots (QDs) and gold nanoparticles (AuNPs) pair, as a mode of donor and acceptor in its uniqueness of energy transfer respectively, is very demanding for the FRET system due to its high efficiency energy transfer and simpler preparation protocols [133]. In the course of proceeding with the detection mechanism, the CdZnTe QDs donors carried on silica were used to stamp an amino group improved aptamer against AFB1 at first, while the thiol group modified complementary DNA (cDNA) of aptamer was used to conjugate the AuNPs (gold nanoparticles) acceptors. The CdZnTe QDs and AuNPs were brought together into close vicinity through hybridization of DNA within aptamer and cDNA, thereby leading to the manifestation of FRET during the aptasensor fabrication. The FRET process was disturbed by the specific binding between aptamer and AFB1 when incubated with AFB1, thus leading to the successive fluorescence retrieval of CdZnTe QDs. When the concentration of AFB1 was up to 100 ng·mL^−1^, the concentration of AFB1 attained a maximum fluorescence recovery since the intensity of the fluorescence augments with the increase in concentration of AFB1. This could be explained by the fact that the higher the existence of AFB1 in the test sample, the larger the number of aptamers inclined to conglomerate with AFB1 to prompt the release of cDNA-AuNPs into the bulk solution, resulting in a considerable fluorescence recovery. Over the wide range of 50–100 ng·mL^−1^, a notable linear relationship between the fluorescence intensity and the concentration of AFB1 was achieved upon normalization. The detection limit was calculated to be 20 ng·mL^−1^ based on S/N = 3.

**Figure 15:**
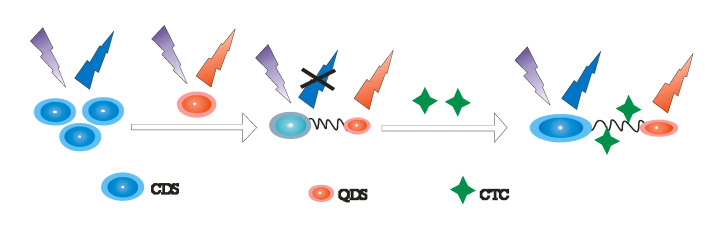
Simplified diagram of fluorescence sensor layout for ratiometric detection of chlorotetracycline.

The treatment of diseases and prevention of bacteria in animal husbandry is achieved by broad spectrum tetracycline antibodies, i.e., chlorotetracycline (CTC) [134]. However, the abuse of CTC in humans accumulates and leads to drug resistance; they require a fast and efficient method to detect them. Until today, measurement of CTC has been determined through fluorescence sensing, which has high superiority of specificity, minimal cost, and remarkable sensitivity [135,136]. Synchronous fluorescence method is adequately used among other fluorescence techniques due to its remarkable resolution, lower scattering of the different wavelengths, and avoidance of interference of some peaks in the conventional fluorescence spectrum. The construction of a dual-mode CTC detection procedure with conventional fluorescence and synchronous fluorescence is therefore necessary. Due to their single emission fluorescence response and the interference of signals by the sensor concentration which obstructs their sensitivity and selectivity, ratiometric fluorescence is however recommended due to its self-calibration ability, and improved selectivity and sensitivity [112,137]. Xiaoping Chen and co-workers [138] synthesized a ratiometric fluorescence sensor dependent on carbon dots (CDs) and copper indium sulphide, zinc sulphide quantum dots shell (CuInS2/ZnS), which detected CTC using conventional fluorescence methods and synchronous fluorescence (Figure 15). This ratiometric sensor showed a good specificity and fast fluorescence response towards CTC among its similar structures. The authors studied the relationship of ΔIg and the concentration of CTC. ΔIg = ICDs/IQDs − I0CDs/I0QDs, where the I0CDs/I0QDs stands for the fluorescence intensities of CDs and QDs in absence of CTC (ΔIg1 stands for the conventional fluorescence, ΔIg2 stands for synchronous fluorescence). Under adequate experimental conditions, the feedback of the sensing technique to CTC was studied by synchronous fluorescence and conventional fluorescence methods. On addition of various solutions with different concentrations of CTC, as the concentration of CTC is intensified the fluorescence of CDs increases, while the fluorescence of CuInS_2_/ZnS (QDs) remain ignorable. There was a favorable linear relationship between ΔIg1 and the concentration of CTC in the range of 1–70 µM with a limit of detection of 0.46 µM, while in the concentration range of 1–50 µM there was an acceptable linear relationship between ΔIg2 and CTC with a limit of detection of 0.36 µM. Comparisons between the outcomes of synchronous fluorescence and conventional fluorescence for the sensing of CTC shows that he linear range of the synchronous fluorescence is smaller than that of conventional fluorescence, but the limit of detection of the conventional fluorescence is larger than that of the synchronous fluorescence. This observation leads to fluorescence response toward CTC (Figure 15). As per the fluorescence sensing of CTC, CuInS_2_/ZnS QDS and CDs could be considered because the shift of signal between ‘off’ and ‘on’ can help with the detection specificity and selectivity [138].

**Figure 16:**
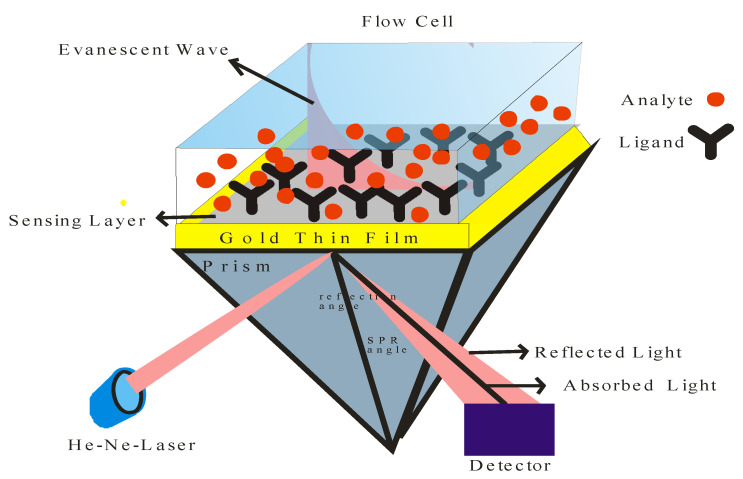
Schematic illustration of SPR setup.

Dopamine (DA) is an important catecholamine neurotransmitter due to the function it performs in the mammalian brain to relate messages from the brain through the nerve impulse [139]. Dopamine controls psychological behavior and the function of the central nervous system, therefore a discrepancy in the normal level of dopamine in the body fluid (130 pM) and urine (5 nM) [140] can result in severe neurodegenerative diseases such as Alzheimer’s disease or schizophrenia [141,142]. The use of RNA aptamer sequence in the detection of DA showed very promising results, however in the DA-RNA binding, the complexity in understanding the electrostatic interactions between the amine group of the DA and the sugar phosphate back bone of the RNA limits this method [143]. In addition to other methods, one of the most progressive technologies being explored for use on bio-molecular interactions is surface plasmon resonance (SPR) [144]. The efficiency of SPR is comparable to other conventional diagnostic techniques. Other advantages of this technique include real time sensing capability, simple preparation and control, no label requirement, high specificity, and sensitivity, which are important advantages over other conventional diagnostic techniques. However, there is a need for the sensitivity enhancement of the determination of low-molecular-weight compounds using this method [145,146]. Faten et al. [147] synthesized a composite of chitosan (CS) and graphene quantum dots (GQDs) film as sensors to detect DA using SPR sensor (Figure 16). GQDs have emerged as one of the carbon-based quantum dots due to their low toxicity, biocompatibility, photostability and relatively high significant quantum yield alongside chitosan, a biodegradable and remarkable molecular biopolymer with numerous amine group, which has been used to prepare the composite (CS-GQDs) suitable for the detection of DA. Generally, the performance of an SPR sensor ought to be analyzed based on its sensitivity and the accuracy. Parameters that should be evaluated include sensitivity, signal to noise ratio (SNR), figure of merit (FOM), detection accuracy, and full width at half maximum (FWHM). With regard to this, the angular shift of resonance reflectance dip (Δθ) is the most important parameter to consider [148]. The Δθ can be computed as the variance between the SPR angle of deionized water (DW) and the SPR angle of different levels of DA solution, while the ratio between the calculated Δθ and the measured concentration of the target could be used to describe the sensitivity of SPR. The change of SPR angle for a given switch in the refractive index (RI) ought to be large as possible to reflect good sensitivity. After the experiment to evaluate the sensitivity, a slope of linear fit was produced which was 0.011 with a correlation coefficient of R^2^ of 0.8174. These values indicate that SPR sensors, in addition to modified CS-GQDs thin film, have a considerable sensitivity. The impressive desirability of DA to the CS-GQDs layer was possibly due to the noncovalent interactions between the carboxyl groups of the GQDs and the hydroxyl, phenyl in DA and diols amine functional groups. Strong π-π interactions, electrostatic interactions, and hydrogen bonding were enabled by the phenyl moiety of DA [113,149,150]. It is presumed that there is an inverse proportionality between the FWHM of the SPR and the detection accuracy of the SPR sensor. The insignificant value of FWHM shows that the error in defining the SPR angle is minute, which in turn leads to perfect results [151]. The decreased value of FWHM of DW from 2.714° to 2.705° for 1 fM of DA emulation given detection exactness from 0.368 deg^−1^ to 0.369 deg^−1^ revealed that with the level of DA the detection increased. To determine the sensor performance and quantify its precision, signal to noise ratio is another essential parameter to consider. The outcome of the experiment revealed that the SPR values for the proposed sensor increase, and this is mostly caused by the noise reduction of the SPR signals which is a result of the increase in DA concentration. As expected, the variation in RI of the sensing layer instigated by the intensified of the concentration of DA solution enhances the resonance shift [152]. To appraise the sensing performance of the proposed structure towards DA, various reproducible measurements were carried out.

**Figure 17:**
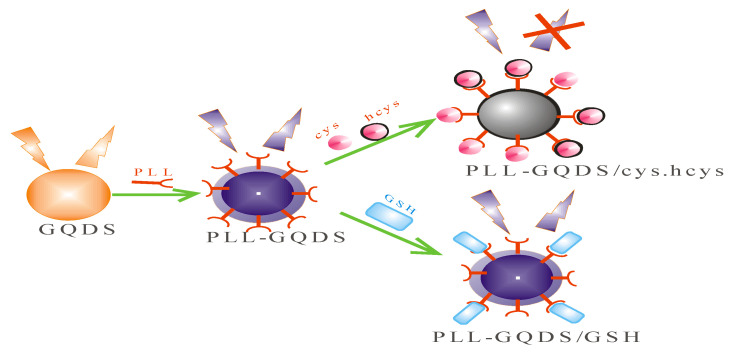
Graphical representation for the detection of cysteine (*cys*) and homocysteine (*hcys*) using PLL-GQDs.

The preservation of physiological processes in human body is mainly achieved by amino acids with thiol groups such as cysteine (cys) and homocysteine (hcys) [153]. A slight discrepancy in the normal level of these biothiols between 240–300 µM for cys and between 5–15 µM for hcys can lead to serious ailments [154,155,156,157]. Therefore, there have been efforts to increase the sensitivity and selectivity of fluorescence sensors to sense and extricate these biothiols. For some years now, various detecting approaches have been developed, however the low detection sensitivity, complex operation procedures, complicated assay procedures, and utilisation of expensive or somewhat toxic materials limit the practical applications of such methods [158]. In view of this, a fluorescence probe with speedy detection, marginal expense, actual time imaging proves to be the most encouraging reasonable method for the detection of biological molecules with thiols [159]. Functionalization with organic groups on the surface offers extraordinary fluorescence behaviors that could distinguish between cysteine (cys) and homocysteine (hcys) [160]. Zamir et al. [161] designed a unique poly-L-lysine (PLL) surface functionalization of graphene quantum dots (GQDs) sensors via a hydrothermal approach using pistachio shells as precursors for the detection of these compounds. A fluorescence sensor (PLL-GQDs) was then made by surface functionalizing GQDS with PLL, which is a decomposable polycationic electrolyte, to advance the sensitivity and selectivity towards cys and hcys (Figure 17). Ultraviolet (UV) absorption and photoluminescence (PL) emission spectra were used to determine the optical features of the quantum dots composite (PLL-GQDs). The agitation as well as the emission of the quantum probe presented an insert, illustrating the composite dispersal under regular light (yellow) and ultraviolet (UV) irradiation (bright blue color) [161]. With the inclusion of cys at ambient temperature in Buffered Phosphate Saline (BPS) pH 7.4, the fluorescence emission intensity of PLL-GQDs showed quenching. At a concentration of 150 nM of cys, the maximum fluorescence quenching was achieved in 2 mL of the fluorescent sensor. With the accumulation of cys in the range of 0–150 nM, the PL emission gradually decreased with a limit of detection of 2.38 nM. The same quenching effect was observed on the fluorescence emission intensity of the probe on addition of hcys at room temperature in BPS with pH value at 7.4. At a concentration of 100 nM of hcys, the utmost fluorescence quenching was perceived for 2 mL of fluorescence material. With the accumulation of hcys in the range of 0–100 nM, the PL emission at 525 nm gradually decreased with a limit of detection of 1.94 nM. The selectivity of the probe was examined by recording the PL emission feedback of sensor to a number of concomitant species such as glutathione (GSH) and it was found that interfering molecules had no effect on the fluorescence intensity of the sensor. The collision of molecules of cys and hcys with surface-functionalized PLL-GQDs could be responsible for the quenching perceived by their introduction. Furthermore, the possible judgment for the fluorescence quenching could be attributed to the non-radiative electron transferal from the excited state of the probe to the cys and hcys [161].

### 2.3. Compounds Used as Sensors in the Detection and Removal of Bisphenol A (BPA) from the Environment

A novel composite of MOFs, electroreduced carbon dots (ErCDs) and gold nanoparticles (AuNPs) was synthesized by Zhan [162] for the sensing and electro-analysis of bisphenol A. The composite of MOFs (AuNPs-ErCDs-MOFs) was synthesized via in situ synthesis by one pot electrodeposition method. This electrodeposition was due to the fact that the electronic transmission capacity of the MOFs composite could be enhanced effectively by AuNPs, ErCDs, and AuNPs-ErCDs, which in turn cause the MOFs to be a good conductive agent, then increase and improve its performance as sensors. When the proposed MOFs composites (AuNPs-ErCDs-MOFs) were used in the sensing and detection of bisphenol A (BPA), the fluorescence of the composite was quenched in the linear range of 7.0 × 10^−8^ mol/L to 5 × 10^−7^ mol/L and 5 × 10^−7^ mol/L to 1.3 × 10^−6^ mol/L for BPA with a limit of detection of 32 nmol/L [162].

Xui et al. [163] used a florescence resonance energy transfer (FRET) sensor centered on nanomaterials up-conversion to determine the presence of pollutants in salty foods. This FRET technique was developed to detect bisphenol A, a common pollutant/contaminant in high-salt foods. The assembly of aptamer improved up-conversion of nanomaterials (DNA2-UCNPs) and complementary DNA-functionalized metal–organic framework (DNA2-MOFs) that retained absorption at similar wavelengths. BPA aptamer for the target BPA signal transduction was used for the performance via the combative identification between the complementary DNA sequences and the BPA analyte in high-salt solution. The water molecules reinforce the MOF to be able to sense and adapt to the high-salt sample and also to steadily enhance the MOF. The composite of MOF (MOF-UCNPs) assembly is highly sensitive for the detection of BPA. Due to its stability under NaCl solution, the MOF acts as a quencher for the FRET assay for BPA to display excellent performance. The MOF exhibited an absorption band at 500–700 nm, an absorption that matches the emission spectrum of UCNPs and the absorption spectrum with the MOF that results in substantial quenching efficiency of approximately 90%. The perfection of the colloidal firmness of the nanosensor was due to the bulky free hydroxyl (OH) groups around the assembly of the MOF. BPA analyte detection showed a limit range of 0.1–100 nM with a low detection limit of 340 mM NaCl food sample [163]. Summary of some of the sensors that have been developed for the detection of bisphenol A is presented in Table 3.

Bisphenol compounds, which are endocrine-disrupting compounds, have been confirmed to be of severe danger to the natural ecosystem and human beings. The detection and removal of these poisonous compounds is of great significance and importance and still remains a huge challenge. Yang et al. successfully designed a Ga-MOF (gallium metal–organic framework) compound by evaluating its fluorescence at a certain and explicit wavelength, which can be employed for the sensitive and selective detection of bisphenol compounds. The Ga-MOF was synthesized by a one-step reaction of Ga(NO_3_)_3_·5H_2_O and 1,2,4,5 benzene tetracarboxylic acid (H4BTC) in a suitable solvent. The careful selection of the gallium metal salt made this MOF sensor have high sensitivity, anti-interference ability and a synchronous fluorescence enhancement and quenching effect on bisphenol compounds. This sensor interestingly represents specific fluorescence behavior of ‘turn on’ and ‘turn off’, which is due to the precise selectivity of the organic linker. The proficient process for evaluating the exposure levels of bisphenols compounds is executed through the Ga-MOF chemical sensor. The Ga-MOF not only exhibits good sensing competency with the detection limit as low as 26.36 nM for the distinctive bisphenol compound but also exceptional selectivity toward the bisphenol on the fluorescence change of ‘turn on’ at 320 nm and a ‘turn off’ at 382 nm. This fluorescence range is due to hydrogen bonding and the π-π stacking interaction between the Ga-MOF host and adsorbed bisphenol molecules. The sensor has an excellent reusability after usage [164].

A direct, rapid, and sensitive electrochemical sensor for bisphenol A (BPA) was established by Chen et al. on a stable three-dimensional porous silicon carbon gold (3D PS-C-Au) electrode. The 3D PS-C-Au preparation was achieved by electrochemical imprint of a nano-gold sputter layer, carbonizing layer of poly furfuryl alcohol (PFA) and single-crystal Si wafers. The nano-gold was vanished by sputter on a 3D porous silicon filer. The stability of the 3D PS-C-Au was scrutinized by real time examining of effective optical thickness (EOT) of the film with an ophthalmic spectrometer in different pH of PBS solutions for 5 h and suitable electroactivity for BPA. The 3D PS-C-Au electrode sensor displays a sound electroactivity, wide linear range from 5.0 × 10^−9^ mol/L to 1.0 × 10^−5^ mol/L of BPA with a low limit of detection of 3.5 × 10^−9^ mol/L (S/N). A recovery ranges from 82.3% to 104.10% was observable by the newly developed method when applied in real time. The increased performance for BPA detection could be attributed to the electrostatic adsorption features of the three-dimensional porous silicon carbon gold to BPA and wide internal surface area. The 3D PS-C-Au sensor has a prodigious potential application in environmental safety field and in food due to its good optical and environmental electrochemical characteristics [167].

**Figure 18:**
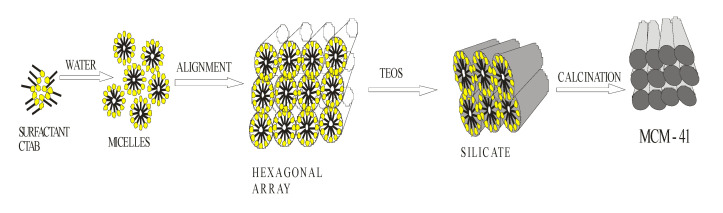
Synthetic scheme for MCM-41 materials.

Fengran et al. [168] engineered a sensitive and selective sensor for BPA which is an evolving pollutant that causes poisonous effects such as causing cancer or disrupting the endocrine system. These authors synthesized a class of mesoporous silica sieve carbon molecular-41 (MCM-41) and then used it to formulate an electrochemical sensor for BPA. In this research the investigation of the electrochemical behaviors of BPA with different electrochemical sensors was carried out. The other sensors were silica gel, carbon nanotubes, graphite and activated carbon. MCM-41 was synthesized using cetyltrimethylammonium bromide (CTAB) as the model. The preparation of a solution of CTAB in NaOH was carried out and it was stirred at 298 k; after that, to give a gel mixture of molar composition of 1:0.25:0.1 to SiO_2_:NaOH:CTAB, respectively, the silica source was added into the solution under stirring. The mixture was precipitated after 30 min of stirring at 298 k and heating at 343 K for 24 h. The precipitate was then dried at 80 °C overnight to remove the CTAB and the MCM-41 was obtained [165]. The electrochemical sensor MCM-41 was then synthesized by, firstly, mechanically mixing the precipitate of MCM-41 sensor obtained with graphite powder and paraffin and, secondly, pressing the carbon paste obtained into the end cavity of the sensor body (Figure 18) [169]. After a thorough sensing study with other electrochemical sensors, and after investigation of the comparison, MCM-41 was found to display a tremendous response signal to the detection of BPA. This wonderful performance of the mesoporous carbon molecular sieve MCM-41 was due to its highly efficient ability, large surface area and other accumulated features compared to other electrochemical sensors. The effect of sensor configuration, pH assessment, and accumulation of time was examined. The detection of the endocrine disruptor BPA was on a linear range from 2.2 × 10^−7^ mol/L to 8.8 × 10^−5^ mol/L with a limit of detection evaluated to be 3.8 × 10^−8^ mol/L.

**Figure 19:**
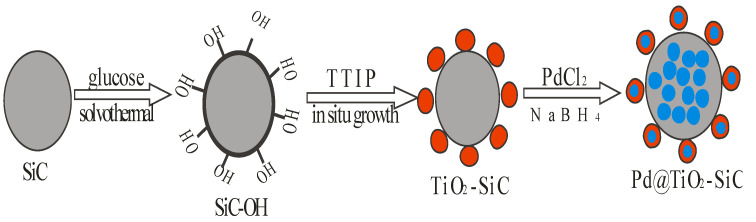
Design and synthesis of Pd@TiO2-SiC nanohybrids.

A titanium dioxide-silicon carbide (TiO_2_-SiC) nanohybrid with an improved electrochemical functioning was synthesized through a simplistic generic in situ development approach. Via a chemical reduction reaction method, Long Yang et al. [170], through the use of polyethylene glycol and sodium citrate as the dispersant and steadying agent respectively, prepared a monodispersed ultrafine palladium nanoparticle (Pd NPs) with size 2.3 nm on the surface of TiO_2_-SiC. The composite (Pd@TiO_2_-SiC) improved glassy carbon electrode (Pd@TiO_2_-SiC-GCE) was synthesized for the simultaneous measurement of hydroquinone (HQ) and bisphenol A (BPA) (Figure 19). The authors realized that based on the admirable properties of palladium nanoparticles, the composites (Pd@TiO_2_-SiC-GCE) had electrochemical ability greater than that of SiC and TiO_2_-SiC in the exposure of the HQ and BPA [171,172]. To simultaneously compute HQ and BPA, distinctive pulse voltammetry was successfully employed within the linear range of 0.01–200 µM under ideal conditions. The detection limit values of Pd@TiO_2_-SiC nanohybrid nanoparticles for HQ and BPA were 5.5 nM and 4.3 nM, respectively. With the introduction of 10% ethanol to the buffer solution, there was an improvement on the selectivity of the electrochemical sensor Pd@TiO_2_-SiC-GCE.

Xiuli et al. [173], developed a novel electrochemical method for sensing of bisphenol A (BPA). The new and suitable electrochemical sensor centered on stacked graphene nanofibers (SGNF) with gold nanoparticle (AuNP)-improved glassy carbon electrodes (GCE). To form a homogeneous dispersion, 10 mg of SGNF was dissolved in 10 mL of deionized water and then sonicated for 1 h. With the use of the microsyringe, 5 µL of the dispersion was dropped into the as pre-treated GCE follow by a sun dried for 8 h in air. Into a 25 mM chloroauric acid solution having 0.1 M sodium sulphate solution as electrolyte, the obtained SGNF/GCE was washed and immersed and there was a deposition of gold nanoparticles. The AuNPs/SGNF/GCE obtained were then compared to SGNF/GCE and AuNPs/GCE electrodes [166,174]. For the oxidation of BPA, an electrocatalytic role was detected when an AuNPs/SGNF electrode was used and there was a significant decrease in the overpotentials of BPA and a remarkable increase in the peak current compared with bare GCE. On AuNPs/SGNF reformed electrodes, the electrochemical oxidation of BPA was a progression of four electrons and four protons as calculated through the charge transfer coefficient (ɑ) and the electron transfer number (n). The operational surface area of AuNPs/SGNF/GCE improved by about 1.7-fold compared to the other electrodes. When linear sweep voltammetry was applied, an enhanced linear relationship between the concentration of BPA and the peak current was attained in the range of 0.08 µM to 250 µM with a limit of detection of 3.5 × 10^−8^ M. Exceptional analytical performance, extraordinary sensitivity, outstanding reproducibility, and long-term stability were perceived by the AuNPs/SGNF/GCE.

Mesoporous carbon (MC) and N-modified mesoporous carbon (NMC) were synthesized by Fang et al. [175] and used as an easy prototype method for the detection and removal of bisphenol A (BPA) through mechanism of adsorption. This adsorption was carried out by a batch and vibrant system and was equated with the marketable activated carbon (AC) [176] by dissolving 2.2 g of F127 (poly (ethylene glycol)-block-poly (propylene glycol)-block-poly (ethylene glycol) diacrylate) in 9 mL of ethanol. The mesoporous adsorbents were prepared and aroused at ambient temperature until totally dissolved. A translucent bright solution was then obtained by adding 2.2 g of resorcinol. Afterward, the mixture was agitated for 36 min after the addition of 9 mL of HCl (3 mol/L) and 0.13 g of melamine. Then, a colloid body was molded after the drop of 2.4 mL of formaldehyde solution into the mixture and stirred for 50 min. The formation of an orange-yellow solid was obtained after the polymerization of the cured colloid in a vacuum oven at 100 °C for 24 h. After that, the solid was roasted in a tube furnace and the temperature of the solid was raised to 350 °C with 1 °C/min rate for 2 h. Then, for another 3 h there was an increase in the temperature from 350 to 800 °C at 2 °C/min rate. The nitrogen-modified mesoporous carbon (the black solid) was then the final product. With the exception of melamine modifier, the preparation of an unmodified mesoporous carbon proceeded. The precise surface area and pore size of MC and NMC were 68.7 m^2^/g and 4.06 nm, and 579.6 m^2^/g and 4 nm, respectively. The original concentration of BPA was positively correlated to the equilibrium adsorption capacity of the sensors, but the adsorbent dosage was negatively correlated to the equilibrium adsorption of the sensors. BPA adsorption on MC and NMC fit well into the pseudo-second order kinetic model and Freundlich isotherm model [177]. The increase in the electronegativity was due to the N–H bond and the decrease in the specific surface area was caused by C–N bond in NMC. These variations make the adsorption favorable, exothermic, and impulsive at lesser temperatures, which facilitates the adsorption of BPA on the adsorbents. Both synthesized adsorbents exhibited an excellent performance for the detection of BPA compared to the activated carbon and also stable recycling performance.

Zhang et al. [178] synthesized a functionalized single wall carbon nanotube (f-SWCNT) carboxylic, a functionalized poly 3,4-ethylenedioxythiophene (PC4) complex, and a modified glassy carbon electrode (GCE) for the voltammetry determination of bisphenol A (BPA). The electrochemical technique was applied to study the performance of BPA at the surface of the reformed electrode. Before the electrodeposition, the modified electrode, which consisted of 0.05 µm alumina, was employed to carefully polish the GCE. Abrasive paper was used to polish the platinum wires; water and absolute ethanol were employed to cleanse it before respective utilization. Then, 0.02 mol/L of (functionalized ethylenedioxythiophene carboxylic group) C4-EDOT-COOH, 0.05 mol/L of lithium perchlorate (LiClO_4_), and 0.05 mol/L of sodium dodecyl sulphate (SDS) in an aqueous micellar solution were employed to electrosynthesize PC4 film at a uniform potential of 0.94 V at room temperature with 70 s as the deposition time. The electrolyte and monomer, which were impurities, were then washed recurrently with distilled water after PC4 film-modified GCE was obtained. To obtain a 1.0 mg/mL suspension of SWCNT, f-SWCNT was disseminated in double-distilled water. The modified electrode f-SWCNT/PC4/GCE was then obtained after 5 mL of the suspension (f-SWCNT) was dropped on the PC4/GCE surface, then dried under an infrared lamp [179,180,181,182,183,184]. At 0.623 V in PBS (0.1 mol/L pH 7.0), a noticeable catalytic activity toward the oxidation of BPA was observed by the electrode from the cyclic voltammetry’s results with a distinct anodic peak. The surface of the 3D network composite was the cause of an enhanced and good adsorption of the analyte. Under an improved condition and a range between 0.099 and 5.794 µmol/L, BPA concentration is proportional to the oxidation peak current with a limit of detection of 0.032 µmol/L (S/N = 3) with a regression coefficient of R^2^ = 0.9989. The excellent electrocatalytic functioning of the f-SWCNT/PC4/GCE and the enhanced conductivity of PC4 lead to the observed performance. The excellent stability and good conductibility were well established.

Elhassan et al. [185] synthesized a novel framework of nanoparticles from NiO, ZnO and NFe_3_O_4_ through hydrogen bonding to form N-NiO@NFe_3_O_4_@N-ZnO composites which are low-cost materials. The structural characterizations confirmed the existence of the three metal oxides. The morphological characterization shows the mean particle size of the nanocomposite to be in the range 30–50 nm. The structural and morphological properties of the as-prepared MOF-nanocomposites were confirmed using Fourier transform infrared (FTIR), powder X-Ray diffraction, thermogravimetric analysis, and transmission electron microscopy [186,187,188]. This new nanocomposite was used as an efficient wastewater remediation for atrazine (ATZ) and bisphenol A (BPA). The nano adsorbent composite N-NiO@NFe_3_O_4_@N-ZnO was fabricated by mixing the same weight (2.0 g) of each metal oxide, N-NiO, N-Fe_3_O_4_, and N-ZnO. To each mixture of nano-metal was added 60 mL of absolute ethanol. The solution was then stirred for 90 min at 50 °C, followed by the evaporation of excess ethanol by heating it for 15–20 min at 70–80 °C. To obtain the final metal oxide framework N-NiO@N-Fe_3_O_4_@N-ZnO, there was an irradiation of the produced paste in a microwave oven in 15.0 mL deionized water for 15 min with intervals of about one minute per each 90 s [189,190,191,192]. The maximum and optimum removal conditions were attained after controlling different experimental parameters: the pH of the mixture, the interaction time, the adsorbent quantity, and the initial concentration of the adsorbate. Finally, 100 g of the adsorbent at pH 5 and the reaction contact time 80 min were the optimum and maximum parameters for 92% of ATZ and 96% for BPA removal from wastewater. This adsorption and removal of these pollutants have been investigated to follow the pseudo second order kinetics model. Langmuir adsorption isotherm best fit the adsorption equilibrium of this experiment.

### 2.4. Metal–Organic Framework Quantum Dots Composites as Sensors for Environmental Pollutants and Other Compounds

Siqi et al. [193] described a novel approach of incorporating the advantages of fluorescent nanomaterials and metal–organic frameworks to improve a unique fluorescent composite for sensor applications. A binary carbon quantum dot with formidable quantum producing nanoparticles with blue emitting color was embedded into a luminescent metal–organic framework (LMOFs: zeolitic imidazole framework ZIF-8). A simple one pot technique via a facile in situ embedding strategy was employed for the synthesis of CQDs@ZIF-8. The mark unit in the composite is ZIF-8 while the signal transmission unit in the apertures of the ZIF-8 framework remains CQDs. Beyond the uniform size, the as-synthesized CQDs@ZIF-8 composite also shows exceptional fluorescence. More significantly, the CQDs@ZIF-8 composite possesses suitable thermal firmness, fluorescent steadiness and demonstrates high precision, and anti-restriction performances for dopamine discovery. The as-prepared CQDs@ZIF-8 composite generates fresh sensing operation when compared with CQDs or with ZIF-8 separately; the detection of dopamine by the composite CQDs@ZIF-8 was favored over a wide linear concentration range of 0.1–200 μM with a limit of detection (LOD) of 16.64 nM [193].

A new and easy scheme of a fluorescence detection mechanism based on metal–organic framework doped with sulfur quantum dots (SQDs) (SQDs@MOFs) was used to sense and detect Cr(VI)/Cr(III) (Cr_2_O_7_^2−^/Cr_2_O_4_^2−^) by 194. Yanqiu et al. [194]. The composite demonstrated outstanding potential as a chemical detector because of its blue-light-emitting fluorescence, stability in water medium and an excellent quantum yield (68%). A resilient and steady blue light emission on the composite was observed due to the successful doping of SQDs on Uio-66-NH_2_. Over a wide range far below the threshold concentration proposed by WHO, the composite sensor exhibits a high detection with the limit of detection of 0.16 µM for Cr(VI) and 0.17 µM Cr(III). The linear filter effect (LFE) was used to propose the detection procedure for Cr(VI) because of its usual high accuracy and quick response. A high recovery rate of about 97.7% was obtained in real water samples with spiked Cr_2_O_7_^2−^ ions that further emphasized its potential in practical applications [194].

Xin et al. used zinc sulfide quantum dots and metal–organic frameworks (ZIF-8) as precursors [195] for the synthesis of QDS/MOFs hybrid material. The as-integrated composite exhibits a magnificent fluorescent feature for detecting ions with an orange red-emitting peak. In buffer solution of Co^2+^ and Cr_2_O_7_^2^ ions, the fluorescence of this composite was selectively quenched, there was also a buffer system with good anti-obstruction of the fluorescence quenching of the composite when successfully applied to the serum of human albumin (HAS). Over a wide linear range of study, the limit of detection of these ions by the composite was 0.27 μM for Co^2+^ and 0.22 μM for Cr_2_O_7_^2^. Under a 254 nm UV-lamp irradiation a color change from orange to red to colorless could be detected with the naked eye [195].

**Figure 20:**
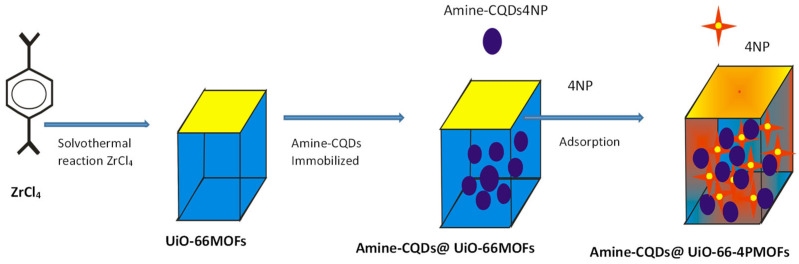
Synthesis of amine-CQDs@MOFs composite and its use as a sensor for 4-nitrophenol.

For the detection of 4-nitrophenol (4-NP), Yang et al. [196] synthesized an amine carbon quantum dot (amine-CQDs) with zirconium-based metal–organic framework (UiO-66) composite fluorescence sensor. A post synthetic method approach (PSMA) was employed to integrate the amine-carbon quantum dot into the framework of the UiO-66. Highly sensitive and selective UiO-66 MOFs was synthesized by mixing zirconium chloride (ZrCl_4_) with terephthalic acid (H_2_BDC) using N, N-dimethylformamide and acetic acid at appropriate ratio. The amine-carbon quantum dots (amine-CQDs) was obtained by dissolving citric acid into deionized water after adding some volume of ethylenediamine. Then, the solution was poured into an auto-clave lined with Teflon and maintained at 200 °C for 5 h. The synthesis of the MOF composite (amine-CQDs@MOF) was then achieved via a PSMA where the MOF (UiO-66 nanocrystal) was submerged into the solution of amine-CQDs for 12 h at room temperature. The product was collected and washed with water followed by ultrasonication. Structural and morphological studies revealed that the amine-CQDs were successfully immersed into the as-synthesized UiO-66 MOFs. The preferential detection of the bond of interaction between UiO-66 and the marked molecule gives these compounds detectable fluorescence signals and this was achieved by the amine-CQDs. The fluorescence-functionalized amine-CQDs@MOFs composite was discovered to be an outstanding scrutinizer for the sensing of 4-NP paralleled to the independent amine-CQDs in solution (Figure 20). This was achieved due to the adsorption and agglomeration of the desired molecule in the openings channel of the UiO-66 and the distinctive cooperation between the performing monomer amine-CQDs and 4-NP. Moreover, 4-nitrophenol could be detected and sensed in a large range concentration of 0.01–2.0 µM with a detection limit of 3.5 nM [196].

Zhou et al. [197] developed a new method for a visual enantiomers probe based on quantum dots incorporated into metal–organic frameworks. The quantum dot was prepared from cadmium chloride (CdCl_2_) and trisodium citrate dihydrate, which were liquefied in water followed by immediate addition of mercaptopropionic acid (MPA) after adjusting the pH to 10.5. Then, amounts of Na_2_TeO_3_ and NaBH_4_ were added to the solution. The preparation of the quantum dots was then achieved by heating the solution up to 100 °C in a microwave for 4 h. The quantum dots were separated after centrifuging the solution at 8000 rpm for 15 min. The quantum dots were wrapped inside Zn_2_camph_2_bipy MOF which was synthesized using Zn(NO_3_)_2_, D-camphoric acid and 4,4′-dipyridyl in DMF. This simple method of synthesis is less time consuming with small and homogeneously sized quantum dot metal–organic framework (QDs@MOFs) particles. A visual, convenient, and direct method was used for the sensing application of D-L tartaric acids, D-L dimethyl tartrates, and D-L mandelic acid. A complete quenching of the fluorescence of the composite was achieved only by L-tartaric acid and it was demonstrated both by size and shape of the MOF. Moreover, there was an increase in the quenching extent with the excess percentage of the mixture composed of D-L tartaric acid enantiomers when the mixture was tested with the analyte. D- and L-dimethyl tartrates when added show a decrease in the fluorescence intensity of QDs@MOFs sensor. On the other hand, no evident alteration in the fluorescence pitch of QDs@MOFs was experienced when D-L mandelic acid was added even after 12 h. This implementation of the mechanism could be extended to enantiomer-selective resolution of other enantiomers. With diverse mobile phases, proof of the accomplishment of chiral separation by QDs@MOFs composite was observed for quite a few pairs of enantiomers [198]. For this method, it is expected that by employing assorted dispersant, one distinct MOF could be able to identify several pairs of enantiomers which can trigger quantum quenching.

The enclosure of a remarkable fluorescence quantum produced branched poly-(ethylenimine) capped carbon quantum dots (BPEI-CQDs) into the zeolitic imidazole framework component (ZIF-8), resulting in an outstanding fluorescent QDs@MOFs synthesized by Xiaomei et al. [199]. This composite was manufactured for the sensing and detection of copper metal ions in environmental wastewater. The incorporated BPEI-CQDs into the ZIF-8 not only allowed the composite to maintain an outstanding fluorescence sensing selectivity and performance but also firmly and judiciously agglomerate marked the analyte due to the adsorption properties of the MOFs. The normalized fluorescence spectrum profile and the fluorescence activity of the composite BPEI-CQDs/ZIF-8 and that of the BPEI-CQDs were almost the same, signifying that the visual optical features of BPEI-CQDs in the ZIF-8 and the surface of BPEI-CQDs was not altered. The accumulation effect of the MOFs triggered the intensification of the sensing frequency from the nanosized fluorescence probe. The quenching trait of the fluorescence of BPEI-CQDs and the adsorption of cation features of the zeolite parents have largely influenced the remarkable selectivity and acuteness of the composites BPEI-CQDs/ZIF-8 for Cu^2+^ ions [200]. The wide range of 2 nM to 1000 nM and the insignificant limit of detection of 80 pM have successfully been recorded in the sensing of Cu^2+^ in the ecological wastewater sample. It is therefore envisaged that different MOFs embedded with formidable fluorescence quantum yield nanoparticles such as quantum dots could be applied as effective and efficient sensors for heavy metals in environmental wastewater.

**Figure 21:**
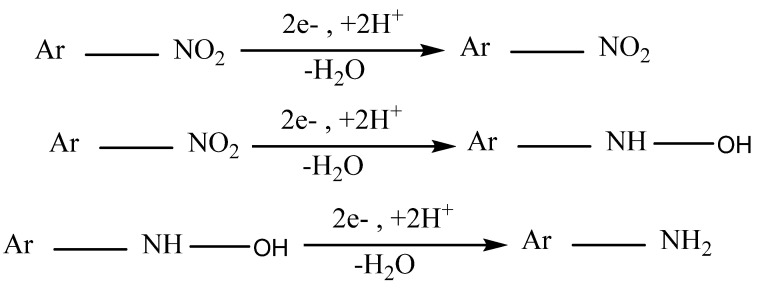
Illustration process of the reaction involved in the formation of aromatic amine from the reduction of aromatic nitro group.

**Figure 22:**
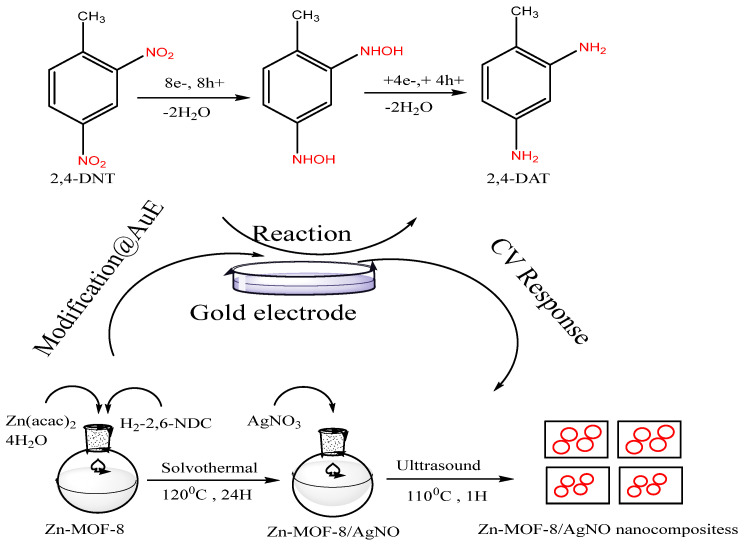
Synthesis of Zn-MOF-8 on a gold electrode and its composite form; 2,4-DNT reduced and depicted by CV.

A highly effective electrochemical zinc metal–organic framework-8 gold electrode modified and silver quantum dots composite (Zn-MOF-8@AgQDs) sensor was successfully synthesized by Sushma Rani et al. [201] for the detection of 2,4-dinitrotoluene. The sensing of 2,4-dinitrotoluene by composite (Zn-MOF-8@AgQDs) modified gold (Au) electrode was achieved via cyclic voltammetry and amperometric methods. The detection of 2,4-dinitrotoluene was achieved via two-step reduction reactions via the following scheme. Firstly, the attainment of a nitroso derivative group from the primary reduction of the nitro group via 2e^−^ and 2H^+^ substitute (removal of water molecules) was carried out. Secondly, the further reduction to hydroxylamine of the nitroso derivative groups via exchange of 2e^−^ and 2H^+^ reaction occurred. Finally, via an elimination of water molecules, the aromatic amine was obtained by the reduction of the hydroxylamine (Figure 21). A total of 12e^−^ and 12H^+^ was required for the whole reaction in obtaining the corresponding amine. The as-synthesized composite was characterized by various characterization methods: transmission electron microscopy (TEM), Fourier transform infrared spectroscopy (FTIR), and powder X-ray diffraction (PXRD). The strong interactions existing between the corresponding formed amines and the surface state of the metal–organic framework enable a proficient detection of the analyte by the composite. Under optimal conditions, the as-synthesized composite exhibits excellent conductivity and good performance of sensitivity and selectivity of 2,4-dinitrotoluene due to various properties possessed by the highly fluorescent silver quantum dots and large surface area of the zinc metal–organic framework (Figure 22). The active linear range of 0.0002 µM to 0.9 µM was recorded and a limit detection of 0.041 µM was observed. The linear sweep voltammetry (LSV) experiment revealed the reduction peak of the analyte at −0.49 V and 0.68 V respectively [201].

One of the micronutrients which serves as a co-enzyme in the biogenetic enzymes in vivo is ascorbic acid (AA) [202]. Pathological and physiological processes are some areas in which ascorbic acid plays a major role. Ascorbic acid shields the existing organism from intracellular strain by being oxidized to dehydroascorbic acid [203]. Due to its ability to be a scavenger for single oxygen, ascorbic acid (AA) stabilizes several compounds including vitamin E and folic acid. However, it has been noted that ascorbic acid is the main cause of several conditions [204,205,206]. Excess of ascorbic acid (greater than 110 µM) in the body leads to urinary disease, diarrhea, and dyspepsia [207,208]. It is therefore necessary to monitor the quantity of ascorbic acid in the body for primal sensing of similar infection. Because of some varieties in interference, such as the reactions in the environs and the concentration around the sensor, which could lead to the obstruction of fluorescence sensors that provide only single emission features [209,210], the proposal of a ratiometric fluorescence (RF) sensor with dual emission signal is of paramount importance [210]. Chen et al. [211] synthesized a stable sensor probe base on cadmium tellurium quantum dots (CdTe) and metal–organic framework (MOF) (UiO-66-NH_2_) and there was an observation of the twin emission apex which was formed when simply conjoining UiO-66-NH_2_ with CdTe owing to the electron transfer from the CdTe to UiO-66-NH_2_. Specific amounts of UiO-66-NH_2_ and CdTe were added together to form the composite sensor of UiO-66-NH_2_@CdTe and then subsequently various concentrations of ascorbic acid were added to the composite to study the fluorescence sensing. The time of incubation was explored, and it revealed a steady time of 20 min before the addition of the ascorbic acid and 10 min after the addition of ascorbic acid. The fluorescence intensity’s ratio of disseminated UiO-66-NH_2_@CdTe was estimated by Io/I where Io = (F_0.430_/F_0.550_); I = (F_430_/F_550_). The fluorescence intensity F_430_ and F_550_ were recorded for UiO-66-NH_2_ and CdTe, respectively, and the results of the blank sample F_0.430_ and F_0.550_ were registered. At the end of the experiment, it was recorded that at 552 nm ascorbic acid improved the emission of CdTe while with the rise in the amounts of ascorbic acid the emission of UiO-66-NH_2_ was quenched. In the concentration range of 200–1200 µM there was a formidable linear affinity between the concentration of ascorbic acid and fluorescence intensity of the sensor and the detection limit of 39.5 µM was recorded. The deviation in the fluorescence color of the UiO-66-NH_2_@CdTe ratiometric fluorescence sensor designates the presence of AA [211]. Summary of binary quantum dots metal–organic framework composite developed as probes for the detection of harmful substances is presented in Table 4.

## 3. Conclusions

Metal–organic frameworks (MOFs) possess many exciting characteristics such as flexibility, ordered crystalline pores and multiple coordination sites, which makes them versatile materials as sensors for environmental pollutants and toxicants. The formation of metal–organic frameworks and coordination polymers were reviewed with the types of ligands. This was followed by a review of the synthetic techniques of quantum dots in aqueous media and their use for the detection of endocrine-disrupting chemicals with summary of various sensors that have been developed to detect bisphenol A. The unique electronic properties and two-dimensional carbon structure of graphene oxide that could be tuned based on the size and shape of the fabricated materials and their incorporation into metal–organic frameworks to develop binary quantum dots/metal–organic frameworks to probe endocrine-disrupting chemicals were examined. In the review, method of synthesis, optoelectronic properties and applications of metal–organic frameworks and quantum dots were examined. The fabrication of metal–organic frameworks/quantum dots as sensors for bisphenol A were reviewed with a focus on how quantum dot properties such as discrete energy level, tunable band gap, narrow emission spectra, high light stability, high sorption, low photo bleaching rates and high quantum yield could be optimized to develop effective sensors. In addition to the high surface area, tunable pore size and volume, adaptability, and flexibility of metal–organic frameworks, their ordered crystalline pores and multiple coordination sites offer various functions and application such as sensing materials for environmental pollutants and contaminants. Bisphenol A has been sensed and detected using various chemical materials such as metal–organic frameworks and quantum dots on a separate level. The review shows that the use of composite materials as sensors for bisphenol A has not been explored and thus it is necessary to developed metal–organic framework quantum dot composites (MOFs@QDs).

## Figures and Tables

**Table 1:** Summary of metal–organic frameworks used for the detection of harmful substances.

No.	MOFs Sensors	Analytes	Linear Range	Limit of Detection	Effect on Luminescence	Ref.
1.	MIL-53-NH_2_	17β-Estradiol (E2)	0.5–1000 nM	0.2–0.3 μM	Quenching	[58]
2.	Co-Ni/MOF	Choramphenicol	1.0 × 10^−13^ M to 1.0 × 10^−6^ M	2.9 × 10^−14^ M	Enhancing	[59]
3.	Ƴ-Fe_2_O_3_/rGO	H_2_S	-	-	Enhancing	[60]
4.	IRMOF@Au-tetrapods	NT-proBNPS	1 fg·mL^−1^ to 1 ηg·mL^−1^	0.75 fg·mL^−1^	Enhancing	[61]
5.	UiO-67-sbdc	GSH	-	107.2 µM	Enhancing	[69]
6.	Co-Ni-MOF	Deoxynivalenol (DON), Salbutamol (SAL)	0.001 to 0.5 ng·mL^−1^	0.05–0.30 pg mL^−1^	Enhancing	[73]
7.	IRMOF-2-X	PhNO_2_	-	-	Quenching	[79,80,81]
8.	Zn_2_(oba)_2_(bpy)^.^	Nitroaromatics	-	-	Quenching	[89]

**Table 2:** Summary of quantum dots used for the detection of harmful substances.

No.	Quantum Dots Sensors	Analytes	Linear Range	Limit of Detection	Effect on Luminescence	Ref.
1.	Graphene oxide (GO)	Phosphate (Pi)	15–37 µM	0.1 µM	Quenching	[93]
2.	CuInS_2_/ZnS	Copper (Cu^2+^)	0–70 nM	63 nM	Quenching	[99]
3.	AgInS_2_	Glutathione (GSH)	3 × 10^−4^ to 2.5 × 10^−3^ mol·L^−1^	2.8 × 10^−10^ mol·L^−1^	Enhancing	[102]
4.	TDES-CdSe	Uranyl ion (UO_2_^2+^)	10–50 nM	5.7 nM	Quenching	[109]
5.	CdZnTe	Aflatoxin 1 (AFB1)	50–100 ng·mL^−1^	20 ng·mL^−1^	Enhancing	[110]
6.	CDs/	Chlorotetracycline (CTC)	1–70 µM	0.46 µM	Enhancing	[111]
	CuInS_2_/ZnS		1–50 µM	0.36 µM	Ignorable	
7.	CS-GQDs	Dopamine (DA)	-	-	Enhancing	[112]
8.	PLL-GQDs	Cysteine (cys)	0–150 nM	2.38 nM.	Quenching	[113]
		Homocysteine (hcys)	0–100 nM	1.94 nM	Quenching	

**Table 3:** Summary of various sensors/probes used for the detection of bisphenol A as a harmful substance.

No.	Sensors/Probes	Analytes	Linear Range	Limit of Detection	Effect on Luminescence	Ref.
1.	AuNPs- ErCDs-MOFs	Bisphenol A (BPA)	7.0 × 10^−8^ to 5 × 10^−7^ mol/L	32 nmol/L	Enhancing	[151]
2.	MOF-UCNPs	Bisphenol A (BPA)	0.1–100 nM	0.02 nM	Quenching	[152]
3.	Ga-MOF	Bisphenol A (BPA)	320–382 nm	26.36 nM	Enhancing	[153]
4.	(3D PS-C-Au) electrode	Bisphenol A (BPA)	5.0 × 10^−9^ mol/L to 1.0 × 10^−5^ mol/L	3.5 × 10^−9^ mol/L	Enhancing	[154]
5.	MCM-41	Bisphenol A (BPA)	2.2 × 10^−7^ mol/L to 8.8 × 10^−5^ mol/L	3.8 × 10^−8^ mol/L	Enhancing	[155]
6.	Pd@TiO_2_-SiC-GCE	Bisphenol A (BPA)	0.01–200 µM	4.3 nM	Enhancing	[158]
7.	N-modified mesoporous carbon (NMC),	Bisphenol A (BPA)	-	-	Enhancing	[164]
	mesoporous carbon (MC)				Enhancing	
8.	f-SWCNT/PC4/GCE	Bisphenol A (BPA)	0.099–5.794 µmol/L	0.032 µmol/L	Enhancing	[165]
9.	N-NiO@NFe_3_O_4_@N-ZnO	Bisphenol A (BPA)	-	-	Quenching	[166]

**Table 4:** Summary of binary quantum dots metal–organic framework composite sensors/probes for the detection of harmful substances.

No.	Sensors/Probes	Analytes	Linear Range	Limit of Detection	Effect on Luminescence	Refs.
1.	CDs@ZIF-8	dopamine (DA)	0.1–200 nM	16.64 nM	Enhancing	[182]
2.	SQDs@MOFs	Cr (VI),	below the threshold	0.16 μM	Enhancing	[183]
		(Cr_2_O_7_^2−^/Cr_2_O_4_^2−^)		0.17 μM		
3.	Mn^2+^ ZnS@ ZIF-8	Co^2+^		0.27 μM	Quenching	[184]
		human albumin (HAS)		0.22 μM		
4.	amine-CQDs@MOFs	4-nitrophenol (4-NP)	0.01–2.0 µM	3.5 nM	Enhancing	[185]
5.	CdTe@Zn_2_camph_2_bipy	L-tartaric	-	-	Quenching	[186]
		D- and L-dimethyl tartrates				
6.	BPEI-CQDs/ZIF-8	Cu^2+^	2 nM to 1000 nM	80 pM	Quenching	[188]
7.	Zn-MOF-8@AgQDs	2,4-dinitritoluene	0.0002 µM to 0.9 µM	0.041 µM	Enhancing	[190]
8.	UiO-66-NH_2_@CdTe	ascorbic acid (AA)	200–1200 µM	39.5 µM	Enhancing/quenching	[201]
